# Harnessing Phytobiotics for Gut Protection: A Comprehensive Review on the Role of Cangzhu (*Atractylodes lancea*) in Sustainable Animal Husbandry

**DOI:** 10.3390/ani16172789

**Published:** 2026-09-04

**Authors:** Mahmoud Soliman, Cheng Du, Xinyu Liu, Liangwenyao Zhao, Hejian Song, Linli Cheng

**Affiliations:** 1 College of Veterinary Medicine, China Agricultural University, Beijing 100084, China; mahmoud.m.solaiman@agr.kfs.edu.eg (M.S.); b20263050716@cau.edu.cn (C.D.); s20253051264@cau.edu.cn (X.L.); sy20253051274@cau.edu.cn (L.Z.); 2 Department of Animal Production, Faculty of Agriculture, Kafrelsheikh University, Kafr El Sheikh 35111, Egypt; 3 Animal Husbandry Workstation, Aba Tibetan and Qiang Autonomous Prefecture Bureau of Agriculture and Rural Affairs, Barkam 620992, China; 18180810361@163.com

**Keywords:** *Atractylodes lancea*, Cangzhu, phytobiotic, gut health, intestinal barrier, gut microbiota, sustainable animal husbandry, antibiotic alternative, One Health

## Abstract

The global transition to antibiotic-free livestock production, accelerated by antimicrobial-resistance concerns, has intensified the search for alternatives to antibiotic growth promoters, and phytobiotics have emerged as key candidates. Cangzhu, the dried rhizome of *Atractylodes lancea* (Thunb.) DC, is a crude drug of traditional Chinese medicine that has attracted substantial attention as a multipurpose phytobiotic. This review synthesizes current knowledge on the phytochemistry, gut-protective mechanisms, and practical application of Cangzhu in sustainable livestock production. Its principal bioactive constituents are sesquiterpenoids (atractylone, β-eudesmol, hinesol, atractylenolides I–III), polyacetylenes (including atractylodin), polysaccharides, and flavonoids. Reported gut-protective mechanisms comprise upregulation of tight junction proteins (ZO-1, occludin, claudin-1); dual suppression of the NF-κB and MAPK-signaling pathways; activation of the Nrf2–Keap1 antioxidant axis; modulation of the gut microbiota; and TLR4-mediated stimulation of mucosal immunity. In swine, poultry, and ruminant studies, *Atractylodes*-based supplementation has been associated with improved growth performance, a lower incidence of diarrhea, favorable intestinal morphology, downregulated pro-inflammatory cytokines (TNF-α, IL-1β, IL-6), beneficial microbiota shifts, and inhibited pathogens. However, much of the mechanistic evidence derives from rodent and in vitro models, and much of the in vivo livestock evidence from the related species *A. macrocephala* (Baizhu) or multi-herb formulations; direct livestock trials with pure *A. lancea* remain limited for several endpoints, and the responses observed in these heterogeneous studies depend on the formulation, dose, and animal species used, as indicated by the explicit evidence-tier and source-species tags applied throughout the text, tables, and figures. The reported effective inclusion levels should be read as experimental study ranges and not as standardized dose recommendations. Framed within the One Health paradigm, Cangzhu is therefore best characterized as a mechanistically substantiated candidate alternative to antibiotic growth promoters whose adoption requires species-specific dose validation, quality standardization, and commercial-scale economic confirmation.

## Simple Summary

Cangzhu is the dried rhizome of *Atractylodes lancea*, a plant used in traditional Chinese medicine. This review examines whether it can support gut health in farm animals and reduce reliance on antibiotic growth promoters. Studies in pigs, poultry, ruminants, and experimental models suggest that Cangzhu extracts and polysaccharides may strengthen the intestinal lining, reduce inflammation and oxidative stress, support immune responses, and favor beneficial gut microorganisms. These effects have been associated with better growth, feed use, intestinal structure, and lower diarrhea rates in some studies. Readers should note that a substantial part of this evidence comes from laboratory (rodent and in vitro) models or from the related species *Atractylodes macrocephala* (Baizhu) and mixed herbal formulas, whereas direct trials using Cangzhu (*A. lancea*) itself in farm animals remain limited for several outcomes. However, the chemical composition of Cangzhu varies with plant origin and processing, and effective doses are not yet standardized across animal species. The inclusion levels reported in the primary studies therefore represent experimental study ranges rather than validated nutritional or therapeutic dose recommendations. Larger commercial studies, consistent quality-control markers, and economic assessments are needed before widespread use. Overall, Cangzhu is a promising plant-based feed ingredient for more sustainable animal production, but practical recommendations require further, species-specific validation in the target livestock species.

## 1. Introduction

The global livestock industry is facing unprecedented challenges. Animal protein production must rise by 70% in order to feed the world’s anticipated 9.7 billion people by 2050; however, production systems are constrained by the escalating antimicrobial resistance (AMR) crisis [1,2]. Antimicrobial resistance (AMR) is one of the top ten worldwide public health hazards, according to the World Health Organization [3]. Bacterial AMR is directly responsible for an estimated 1.27 million deaths globally in 2019 [4]. The use of antibiotics in agriculture contributes substantially to this crisis, with an estimated 73% of all antimicrobials used globally in food-producing animals [5].

As a result, a worldwide regulatory change was sparked by the European Union’s 2006 ban on AGPs. Veterinary antibiotic consumption fell from 69,292 metric tons in 2014 to roughly 32,500 metric tons in 2021 when China, the world’s largest consumer of veterinary antibiotics, imposed a complete AGP ban in July 2020 [6]. However, these prohibitions have revealed weaknesses, such as a rise in necrotic enteritis in poultry and post-weaning diarrhea in piglets [7]. These outcomes highlight the critical need for long-term substitutes that actively promote gut health.

Phytobiotic plant-derived bioactive compounds incorporated into animal feed have emerged as promising alternatives to antibiotics [8,9]. In contrast to synthetic compounds, phytobiotics exert their effects through multiple bioactive constituents acting on diverse molecular targets, which may reduce selective pressure for resistance development [10]. Phytogenic feed additions have been shown to boost body weight gain by 5–13% in chicken and up to 30% in lambs, according to a recent systematic review. These additives are particularly beneficial under stressful conditions [11].

Cangzhu, the dried rhizome of *Atractylodes lancea* (Thunb.) DC. (Asteraceae), occupies a special place at the nexus of contemporary pharmacological research and traditional ethnomedical knowledge. For more than two millennia, gastrointestinal conditions like diarrhea, bloating, and anorexia have been treated with Cangzhu in traditional Chinese medicine (TCM) [12]. Its titles, “So-jutsu” in Japan and “Khod-Kha-Mao” in Thailand, demonstrate its pan-Asian therapeutic importance [13]. For clarity of nomenclature: throughout this review, “Cangzhu” refers specifically to Mao Cangzhu, the dried rhizome of *A. lancea* (Thunb.) DC, unless otherwise indicated; evidence derived from the closely related *A. macrocephala* Koidz. (Baizhu) or *A. chinensis* (Bei Cangzhu) is explicitly flagged as such in the text, tables, and figures.

The six primary positive effects that were shown were tight junction protein upregulation for intestinal barrier repair, NF-κB suppression for anti-inflammatory activity, Nrf2 activation for antioxidant defense, microbiota composition modulation, immune function enhancement (IgA and sIgA), and growth performance promotion (improved ADG and FCR). The authors’ synthesis is based on Wang et al. [14], Qu et al. [15], Gao et al. [13], and Koonrungsesomboon et al. [16]. The figure was drawn specifically for this review: its content was synthesized and redrawn by the authors from the mechanistic findings reported in the cited sources, and no figure elements were reproduced directly from copyrighted material.

**Figure 1:**
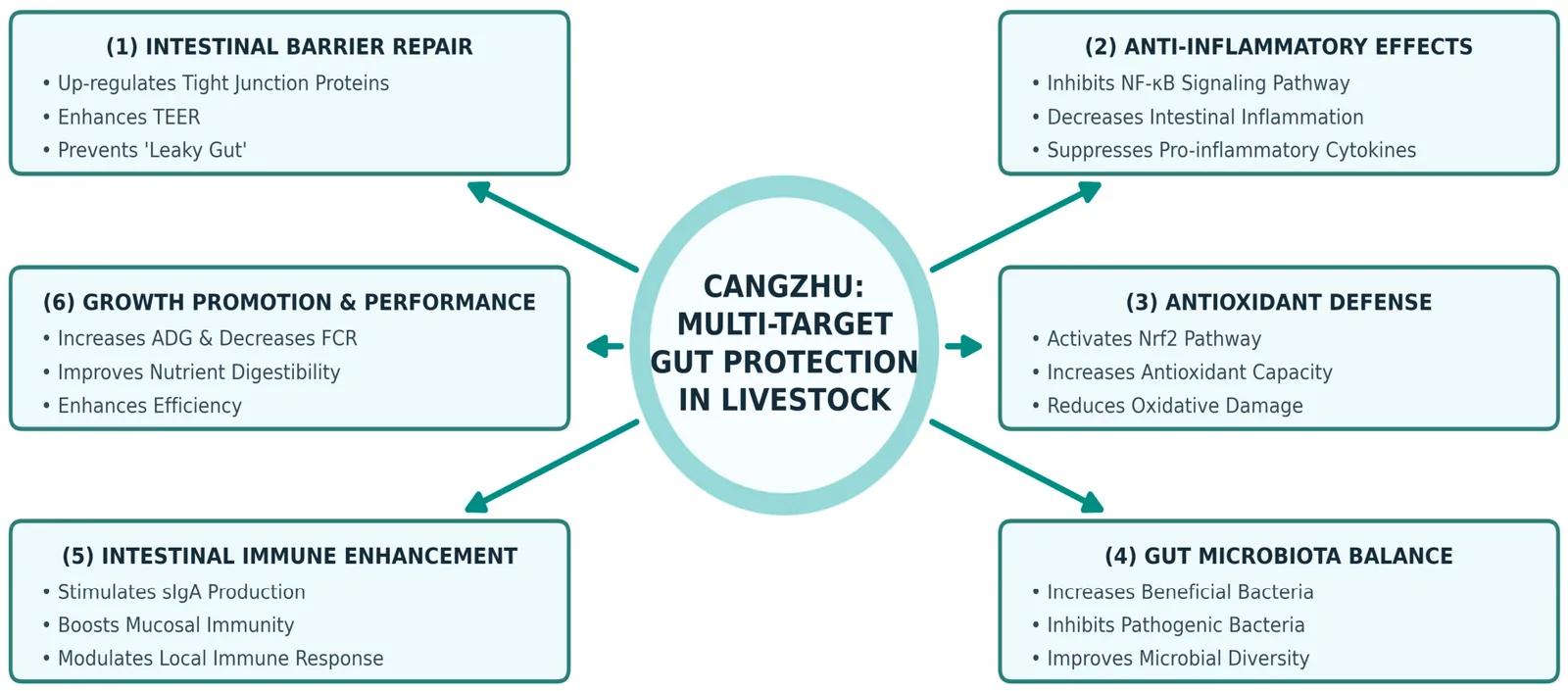
The advantages of Cangzhu (*Atractylodes lancea*) for multi-target gut protection in livestock production.

Despite mounting data including a recent broad overview of the biological functions of *A. lancea* in animal husbandry [13] what remains missing is a mechanistic, gut-centered synthesis that connects molecular mechanisms of intestinal protection with species-specific, dose-resolved application in livestock production. Distinct from that work, this review verifies livestock evidence at the species level (*A. lancea* vs. the frequently conflated *A. macrocephala*, Baizhu), analyzes dose–response across swine, poultry, and ruminants, and frames Cangzhu within AMR-mitigation, One Health, and standardization roadmaps. An overview of these multi-target gut-protective mechanisms is presented in Figure 1. To keep this species-level distinction transparent throughout, the subsequent synthesis tables explicitly identify the Atractylodes species/formulation used in each study together with its evidence tier (direct livestock in vivo, in vitro, or experimental model), and findings derived from *A. macrocephala* or multi-herb formulas are consistently labeled as extrapolated evidence rather than direct *A. lancea* data.

## 2. Materials and Methods: Literature Search and Selection

This article is a narrative review prepared using a structured literature search guided by the PRISMA 2020 statement (Page et al. [17]). The PubMed/MEDLINE, Web of Science, Scopus, and China National Knowledge Infrastructure (CNKI) databases were searched from inception to August 2026 (the final literature update was completed in early August 2026, immediately prior to manuscript submission; accordingly, the review includes studies published in 2025 and 2026, e.g., [13,18,19]), supplemented by Google Scholar and hand-searching of the reference lists of retrieved articles, including the recent overview by Gao et al. [13]. The search strategy combined Atractylodes-related terms with gut-health terms: (“*Atractylodes lancea*” OR “Cangzhu” OR “atractylon” OR “atractylodin” OR “atractylenolide” OR “hinesol” OR “ β-eudesmol”) AND (“gut” OR “intestinal” OR “enteric” OR “microbiota” OR “intestinal barrier”).

Screening and study selection. After removal of duplicates (automated matching in reference-management software followed by manual confirmation), titles and abstracts were screened independently by two reviewers (M.S. and C.D.) against the eligibility criteria below, and candidate full texts were reassessed independently by the same two reviewers; disagreements were resolved by consensus and, where required, by adjudication of a third reviewer (C.L.). For each included study, the following fields were extracted: Atractylodes species and plant part, formulation and dose, animal model or in vitro system, study design, outcome measures, and reported statistics. Given the narrative character of this review and the heterogeneity of the primary literature, no formal risk-of-bias instrument (e.g., ROB 2 or SYRCLE) was applied; instead, evidentiary weight was appraised narratively according to study design, model species, sample size, and whether the intervention used pure *A. lancea*, a related Atractylodes species (e.g., *A. macrocephala*), or a multi-herb formulation. This evidence tier is indicated explicitly in the respective livestock synthesis tables (Section 5).

Peer-reviewed original research articles, reviews, and pharmacopoeial monographs published in English or Chinese were included if they (i) described the phytochemistry or biological activities of *A. lancea* or its constituents; (ii) reported in vitro or in vivo effects on gastrointestinal functions, including barrier integrity, inflammation, oxidative status, immunity, or microbial ecology; or (iii) evaluated Atractylodes-containing interventions in food-producing animals. Records not involving Atractylodes material, lacking gut-related outcomes, or unrelated to animal production, as well as conference abstracts without full text, were excluded.

**Figure 2:**
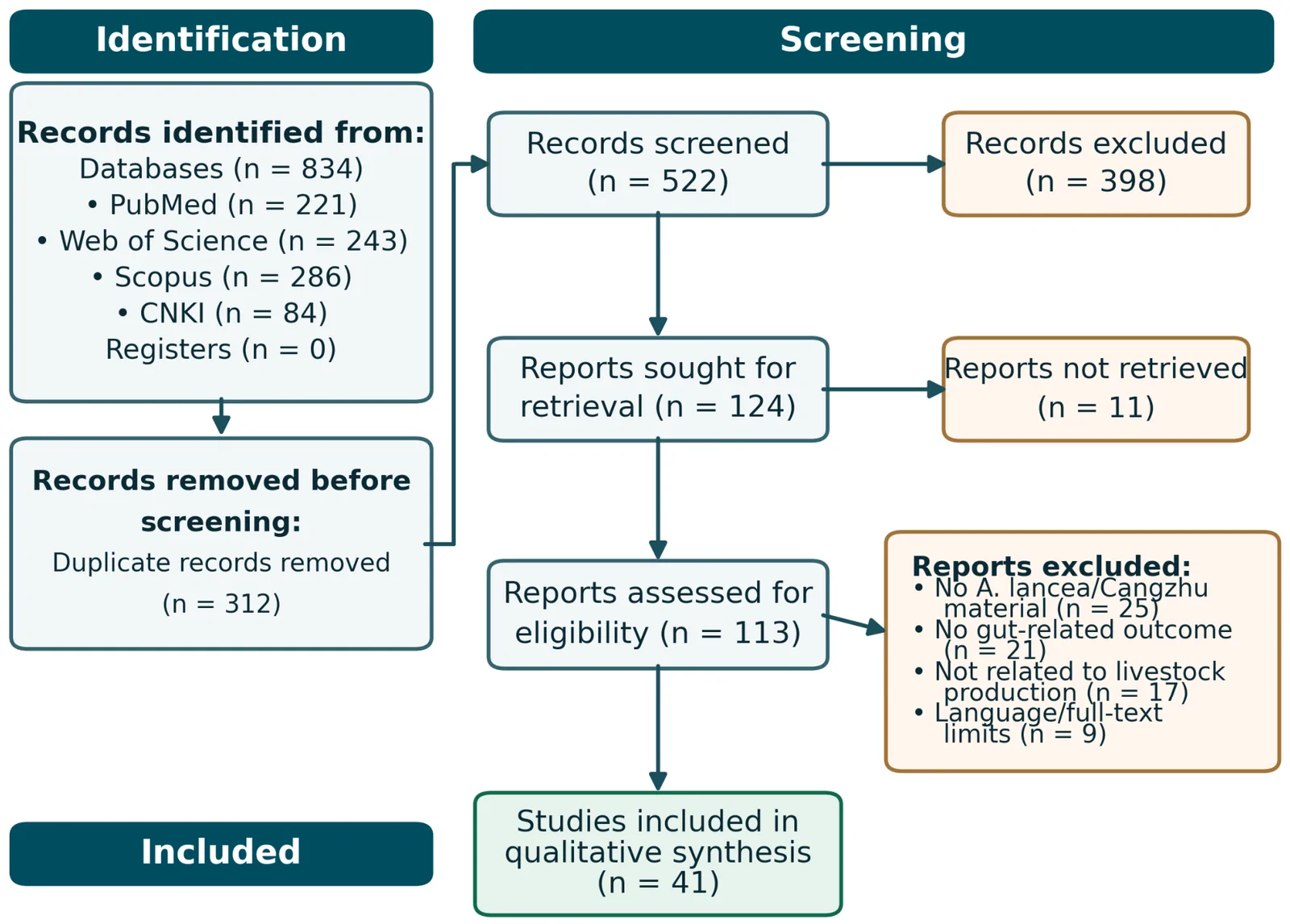
PRISMA 2020 flow diagram of the literature search and study-selection process. Authors’ synthesis based on the PRISMA 2020 statement (Page et al. [17]).

The search identified 834 records (PubMed *n* = 221; Web of Science *n* = 243; Scopus *n* = 286; CNKI *n* = 84). After removal of 312 duplicates, 522 records were screened by title and abstract; 124 reports were sought for retrieval, of which 113 were assessed for eligibility and 72 were excluded (Figure 2). Finally, 41 studies were included in the qualitative synthesis. Given the heterogeneity of study designs, species, and outcome measures, the findings were synthesized narratively; no meta-analysis was performed. Accordingly, subsequent figures are schematic syntheses of the individual cited studies and must not be interpreted as pooled or meta-analytic estimates (see the revised figure captions).

The predominant reasons for exclusion at the title/abstract screening stage were records not involving Atractylodes material (largely pharmacological studies of other botanicals), records without gastrointestinal or gut-related outcomes, records unrelated to animal production or gut health, and conference abstracts without retrievable full text, and records published in languages other than English or Chinese; the complete screening log is available from the corresponding author upon request.

## 3. Botanical Identity and Phytochemical Arsenal of Cangzhu

### 3.1. Botanical Origin and Traditional Context

China, Japan, Korea, and Thailand are home to the perennial *Asteraceae* herbs *Atractylodes lancea* (Thunb.) DC. (Mao Cangzhu) and *Atractylodes chinensis* (DC.) Koidz. (Bei Cangzhu). Their dried rhizomes are the botanical drugs “Mao Cangzhu” and “Bei Cangzhu,” respectively [13,20]. Unless otherwise indicated, “Cangzhu” throughout this review refers specifically to Mao Cangzhu (*A. lancea*); any evidence from Baizhu (*A. macrocephala*) or Bei Cangzhu (*A. chinensis*) is explicitly flagged as such. Its dried rhizome is referred to as “cangzhu” [13,20]. The Chinese Pharmacopeia (2020 edition) states that dried rhizomes must have at least 0.30% atractylodin (C13H10O) [12]. The best Cangzhu is said to be produced in Jiangsu Province’s Maoshan region [20]. The Dabieshan chemotype (higher hinesol and β-eudesmol) and the Maoshan chemotype (enriched in atractylodin and atractylone) are the two main chemotypes that GC-MS metabolomic profiling has identified. The cumulative relative content of the three main actives ranges from 76.19% to 86.11% across developmental stages [18].

### 3.2. Major Bioactive Constituents

**Figure 3:**
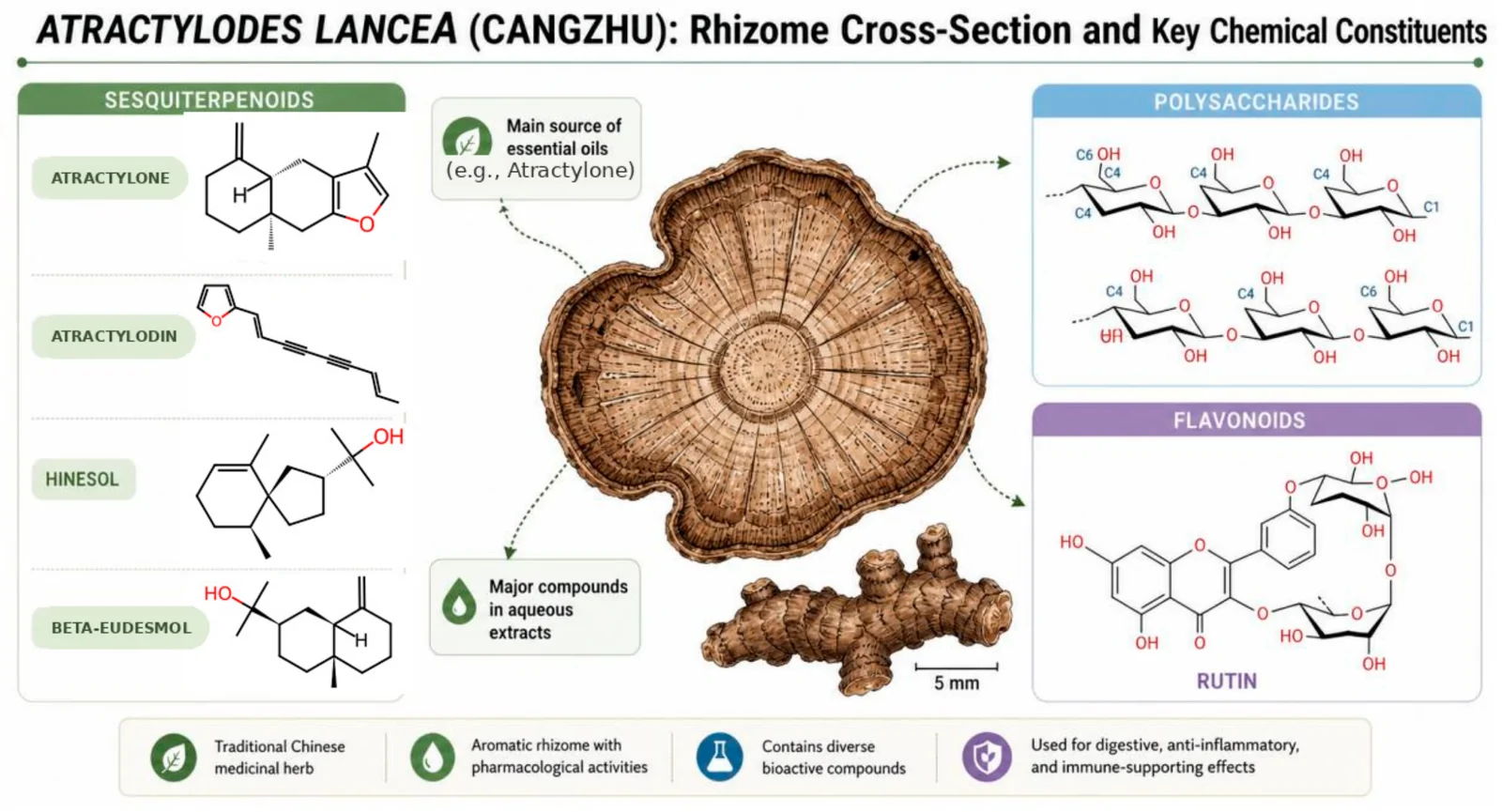
The structural classification of the main bioactive components of the rhizome of Cangzhu (*Atractylodes lancea*).

Comprehensive phytochemical studies have identified a diverse array of bioactive constituents in *A. lancea* rhizomes, as shown in Table 1 and Figure 3 [13,18,20,21]. Because reporting in the primary literature derives from different Atractylodes species and analytical bases, each class in Table 1 is annotated with the species from which the reported value originates, so that *A. lancea* data are not conflated with those obtained from *A. macrocephala*.

The primary bioactive constituents include sesquiterpenoids (atractylone, β-eudesmol, hinesol, and atractylenolides I/II/III), polyacetylenes (including atractylodin and acetylatractylodinol), polysaccharides, flavonoids, and phenolic acids. GC-MS analysis revealed that the cumulative relative content of the three primary actives (hinesol, β-eudesmol, and atractylodin) ranged from 76.19% to 86.11% across developmental stages. The authors’ synthesis was based on Gao et al. [13], Wu et al. [21], and Zhang et al. [18]; the classification of the compounds (and the attribution of atractylodin to the polyacetylenes) was synthesized by the authors from the cited sources, and no elements were reproduced directly from them.

### 3.3. Phytochemical Variability and Standardization Challenges

A key barrier to Cangzhu’s broad usage as a standardized phytobiotic is the vast variance in its phytochemical makeup, which is influenced by botanical species, geographic origin, harvest season, post-harvest processing, and extraction technique [18]. In mice, bran-fried (Fu Chao) polysaccharides (FALP) demonstrated superior therapeutic efficacy at 1.2 g/kg/day compared to raw polysaccharides (SALP) [14]. These data are derived from a rodent model and should be read as preclinical evidence for *A. lancea* [direct: *A. lancea*; rodent model]. A critical step toward quality-controlled applications is the development of validated chemical fingerprinting techniques, such as the GC-MS-SIM approach for the simultaneous measurement of atractylone, hinesol, β-eudesmol, and atractylodin with recovery rates of 97.7–99.7% [22].

## 4. Molecular Mechanisms of Gut Protection by Cangzhu

**Figure 4:**
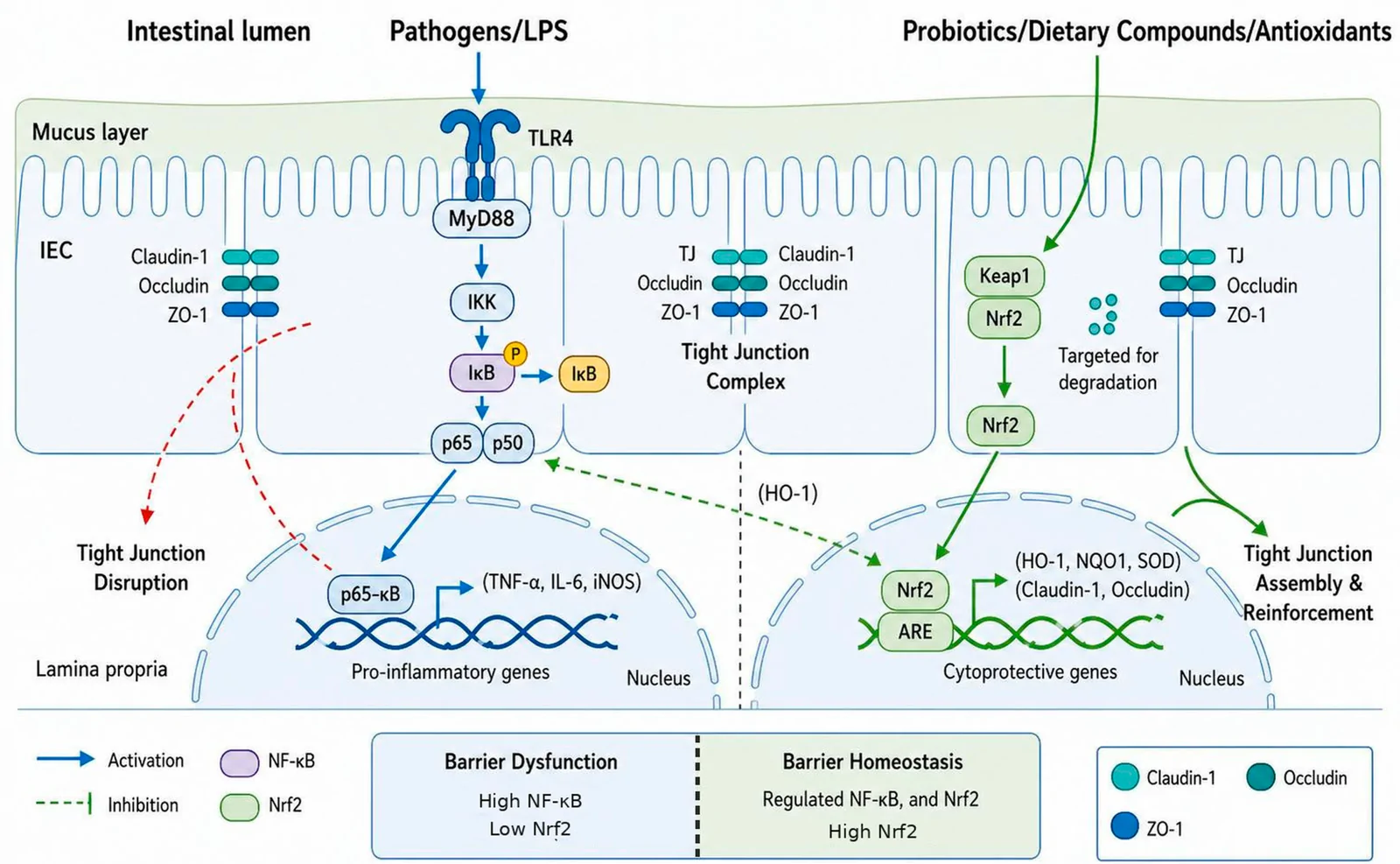
Integrative molecular mechanisms of gut protection by Cangzhu (*A. lancea*). Red dashed arrows indicate processes contributing to tight junction disruption and intestinal barrier impairment.

The coordinated action of several bioactive components on linked signaling pathways is what gives Cangzhu its gut-protective properties (Figure 4). As detailed below, much of the mechanistic evidence originates from rodent models and in vitro systems, with direct demonstration in food-producing animals still emerging; the evidence tier of each finding is therefore indicated throughout this section and in the respective livestock synthesis tables (Section 5). A comprehensive synthesis of the molecular targets, active constituents, and biological outcomes across experimental models is detailed in Appendix A (Table A1).

(A) strengthening the intestinal epithelial barrier by upregulating tight junction proteins (ZO-1, Occludin, and Claudin-1) through inhibition of p38/MAPK-signaling; (B) suppressing the NF-κB pathway, which lowers pro-inflammatory cytokines (TNF-α, IL-1β, and IL-6) and enzymes (iNOS and COX-2); (C) activating the Nrf2-Keap1 antioxidant defense pathway, which increases the transcription of antioxidant enzymes; (D) reshaping the composition of the gut microbiota with more beneficial taxa; and (E) TLR4-mediated immunomodulation, which enhances mucosal immunity through T cell activation and sIgA secretion. The authors’ synthesis was based on Wang et al. [14], Qu et al. [15], Gao et al. [13], and Koonrungsesomboon et al. [16]. The figure was drawn specifically for this review based on the authors’ synthesis of these sources; no elements were reproduced directly from copyrighted material. Red dashed arrows indicate processes leading to tight junction disruption and epithelial barrier impairment.

### 4.1. Reinforcement of the Intestinal Epithelial Barrier

The intestinal epithelium is maintained by tight junction (TJ) protein complexes ZO-1, occludin, and claudins, which regulate paracellular permeability [23]. Compromised TJ integrity is a hallmark of post-weaning intestinal dysfunction and stress-induced enteropathy in poultry [11]. In rodent models and in vitro systems, *A. lancea* extracts and polysaccharides have been reported to increase the mRNA and protein expression of ZO-1, Claudin-1, and Occludin through the inhibition of p38/MAPK phosphorylation [13,14,15] [extrapolated: *A. lancea* in rodent/in vitro models]; to date, direct confirmation in livestock is limited to an *A. macrocephala*-containing herbal combination (GA) that upregulated jejunal ZO-1 and CLDN1 mRNA in broilers [19] [extrapolated: *A. macrocephala* in vivo; see the poultry synthesis table in Section 5.2]. This mechanism involves the direct stimulation of TJ protein gene transcription and, as described for Atractylodes extracts, promotion of mucin secretion by goblet cells [24]. It should be noted, however, that for *A. lancea* specifically, the TJ and mucin (MUC2) evidence is derived predominantly from rodent and in vitro settings; these barrier-enhancing responses are mechanistically plausible in livestock but have not yet been directly demonstrated in food-producing animals with pure *A. lancea*.

### 4.2. Anti-Inflammatory Signaling: NF-κB and MAPK Pathway Suppression

NF-κB and MAPK cascades are the main drivers of intestinal inflammation, culminating in the transcriptional activation of pro-inflammatory cytokines (TNF-α, IL-1β, IL-6) and enzymes (iNOS, COX-2) [25]. NF-κB p65 nuclear translocation is decreased by atractylenolides I and III (in vitro and rodent models) [16,21]. In LPS-stimulated RAW 264.7 macrophages (in vitro), *A. lancea* ethanolic extracts reduced the production of NO and PGE2 as well as the expression of iNOS and COX-2 mRNA [13] [extrapolated: *A. lancea* in vitro]. Atractylenolide II inhibits the phosphorylation of p38, ERK1/2, and JNK [13]. Cangzhu improves intestinal water reabsorption, increases autophagy genes, and decreases pro-inflammatory cytokine release in experimental (rodent) post-weaning colitis models [13] [extrapolated: *A. lancea* in rodent model]. Single-pathway strategies are limited by compensatory activation, which is circumvented via dual-pathway inhibition. These anti-inflammatory findings await direct confirmation in livestock species.

### 4.3. Nrf2-Keap1 Antioxidant Defense Pathway Activation

Under baseline conditions, Keap1 sequesters Nrf2, the main regulator of the cellular antioxidant response. According to Bellezza et al. [26], oxidative stress enables Nrf2 nuclear translocation, which drives the transcription of HO-1, NQO1, GSTA1, GSTM1, and SOD. In LPS-exposed mice, *A. lancea* polysaccharides decreased hepatic MDA levels while raising SOD and GSH-Px levels [13] [extrapolated: *A. lancea* in rodent model]. Atractylodes extracts block p38 phosphorylation while simultaneously promoting Nrf2 nuclear translocation and upregulating HO-1, NQO1, GSTA1, and GSTM1 [21]. Because these pathways are mutually antagonistic, the combination of Nrf2 activation and NF-κB repression is ideal [26]. This results in increased resistance to oxidative stress that is particularly relevant to heat-stressed chickens and early-weaned piglets [13,27], although direct antioxidant measurements in livestock following *A. lancea* supplementation remain scarce and this extrapolation is made cautiously.

### 4.4. Gut Microbiota Modulation and SCFA Metabolism

**Figure 5:**
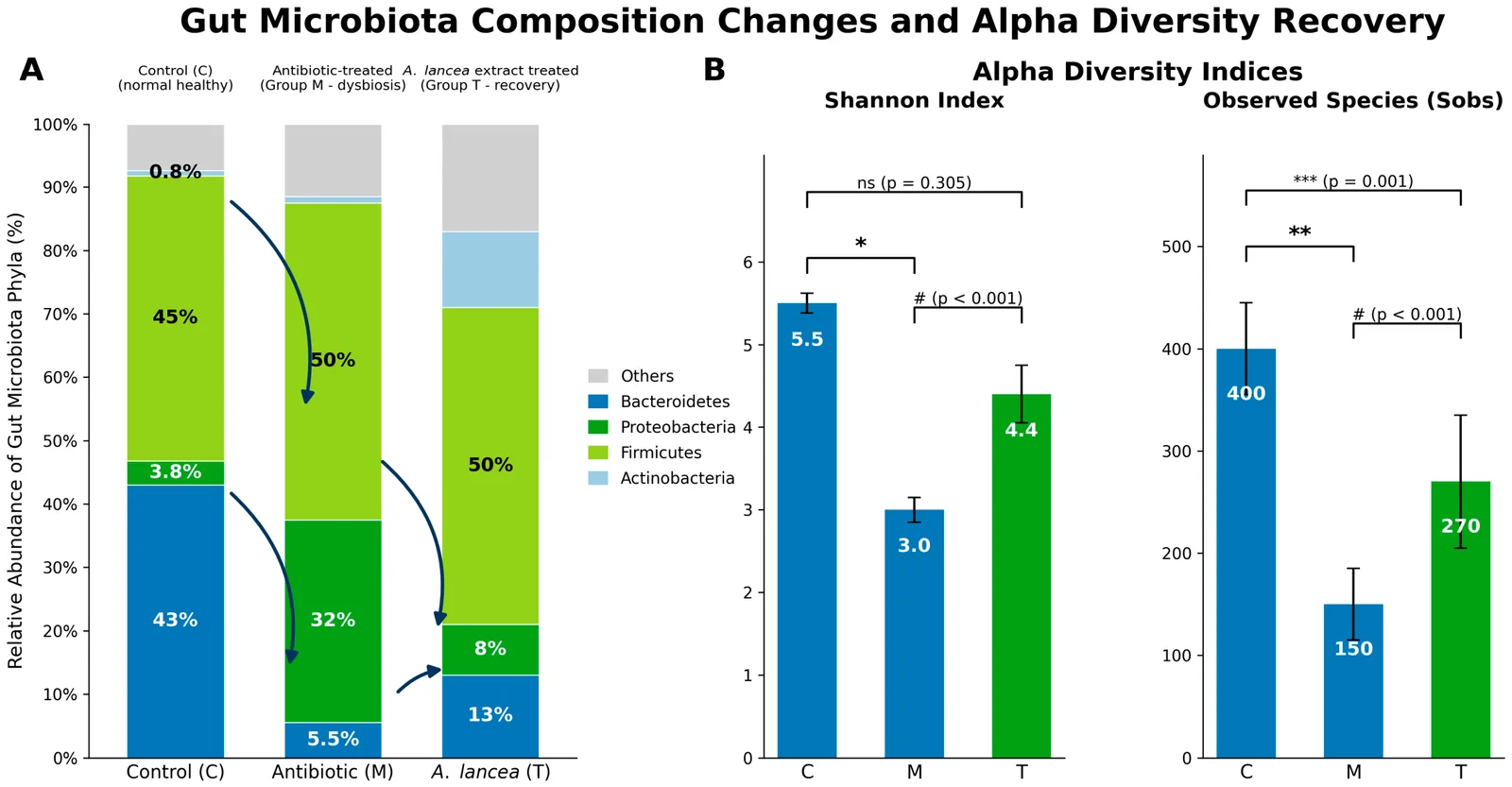
The impact of Cangzhu (*A. lancea* extract) on the microbial diversity and composition of the gut microbiota in a mouse model of antibiotic-induced dysbiosis. (**A**) Phylum-level relative abundance of the gut microbiota (stacked bars) in control mice (C, normal healthy), antibiotic-treated mice (M, dysbiosis; cefradine + gentamicin), and *A. lancea* extract-treated mice (T, recovery): Proteobacteria increased from 3.8% to 32% after antibiotic injection, while Bacteroidetes fell from 43% to 5.5%; *A. lancea* treatment decreased Proteobacteria overgrowth and partially recovered Bacteroidetes (to 13%). Curved arrows in panel (**A**) indicate the direction of the treatment sequence (healthy control → antibiotic-induced dysbiosis → recovery with *A. lancea* extract treatment). (**B**) alpha-diversity indices—the Shannon index and the observed species (Sobs)—which returned to their pre-antibiotic levels. Statistical annotations are those reported in the original study [15]: ns denotes not significant; *, **, ***, and # denote statistically significant differences between groups, with exact *p*-values indicated in the respective panels. At the genus level, Escherichia–Shigella abundance decreased whereas Lactobacillus abundance increased. The authors’ synthesis was based on Qu et al. [15].

Stress-induced intestinal dysfunction is consistently characterized by dysbiosis, which has a significant impact on host nutrition, immunology, and barrier function [28,29]. The effects of *A. lancea* extract on antibiotic-induced dysbiosis in SPF mice were examined by Qu et al. [15] using 16S rRNA sequencing and metabolomics [extrapolated: *A. lancea* in rodent model]. The results showed that *A. lancea* treatment partially restored Bacteroidetes (from 5.5% to 13%), decreased Proteobacteria overgrowth, increased *Lactobacillus*, and restored arachidonic acid and L-methionine metabolism (Figure 5). Butyricicoccus and Muribaculaceae, two bacteria that produce SCFA, were more common in chicken when an Atractylodes-containing herbal combination was used, whereas Desulfovibrio and Campylobacter, two taxa that cause inflammation, were less common [19] [extrapolated: *A. macrocephala*-containing formula in poultry]. The main energy substrate for colonocytes is butyrate, which also suppresses NF-κB activation by acting as an HDAC inhibitor [30]. These compositional observations should be interpreted with caution: 16S rRNA-based profiling reflects relative (compositional) abundance shifts rather than demonstrated restoration of microbial balance, carries well-recognized primer and compositional biases, and does not by itself establish causality (see also Section 7.3). In vitro rumen fermentation of Rhizoma Atractylodis oil improved microbial-protein synthesis and nutrient degradability in batch cultures from Jinjiang yellow cattle [13,31] [extrapolated: *A. lancea* in vitro only]; these rumen findings are, however, restricted to in vitro batch-culture systems, in vivo dose confirmation in ruminants remains limited, and this caveat is likewise flagged in Section 5.3.

Proteobacteria increased from 3.8% to 32% after the injection of antibiotics (cefradine + gentamicin), while Bacteroidetes fell from 43% to 5.5%. Treatment with *A. lancea* extract decreased Proteobacteria overgrowth and partially recovered Bacteroidetes (to 13%). The diversity indexes of Shannon and Sobs alpha returned to their pre-antibiotic levels. At the genus level, Escherichia-Shigella abundance decreased whereas *Lactobacillus* abundance increased. The authors’ synthesis was based on Qu et al. [15]. Note: values are relative abundances from a single 16S rRNA-based mouse study [15]; they are summarized here as mechanistic (preclinical) evidence [extrapolated: *A. lancea* in rodent model] rather than livestock data, and the observed shifts reflect a partially normalized (rather than fully restored) microbial composition.

### 4.5. Immunomodulation and Mucosal Immunity

According to Pluske et al. [32], the largest immune organ in vertebrates is gut-associated lymphoid tissue (GALT). In the related species *A. macrocephala* (Baizhu), rhizome polysaccharides in early-weaned piglets stimulated lymphocyte proliferation, enhanced antibody production, and reduced diarrhea [33] [extrapolated: *A. macrocephala* in vivo], while an Atractylodes-containing fermented formulation at 0.1–0.3% dose-dependently elevated serum IgA, IgG, and IgM [34] [extrapolated: multi-herb formula in vivo]; these findings indicate translational potential for *A. lancea* polysaccharides in weanling piglets [13]. It must be emphasized that these swine immunomodulation data constitute extrapolated evidence obtained with *A. macrocephala* and mixed formulations, not direct *A. lancea* trials. In cyclophosphamide-immunosuppressed mice, *A. lancea* rhizome polysaccharides (ALP) at 1.2 g/kg/day enhanced immune organ indices, blood cell counts, T lymphocyte proliferation, and sIgA production at the cellular level [14] [extrapolated: *A. lancea* in rodent model]. According to Wang et al. [14], microbially produced SCFAs have immunomodulatory effects by upregulating GPR43 expression and triggering the TLR4 signaling pathway. Bran-fried ALP (FALP) had superior immunostimulatory activity in comparison to raw ALP (SALP), suggesting that traditional processing optimizes bioactivity [14].

## 5. Applications of Cangzhu in Livestock Production Systems

**Figure 6:**
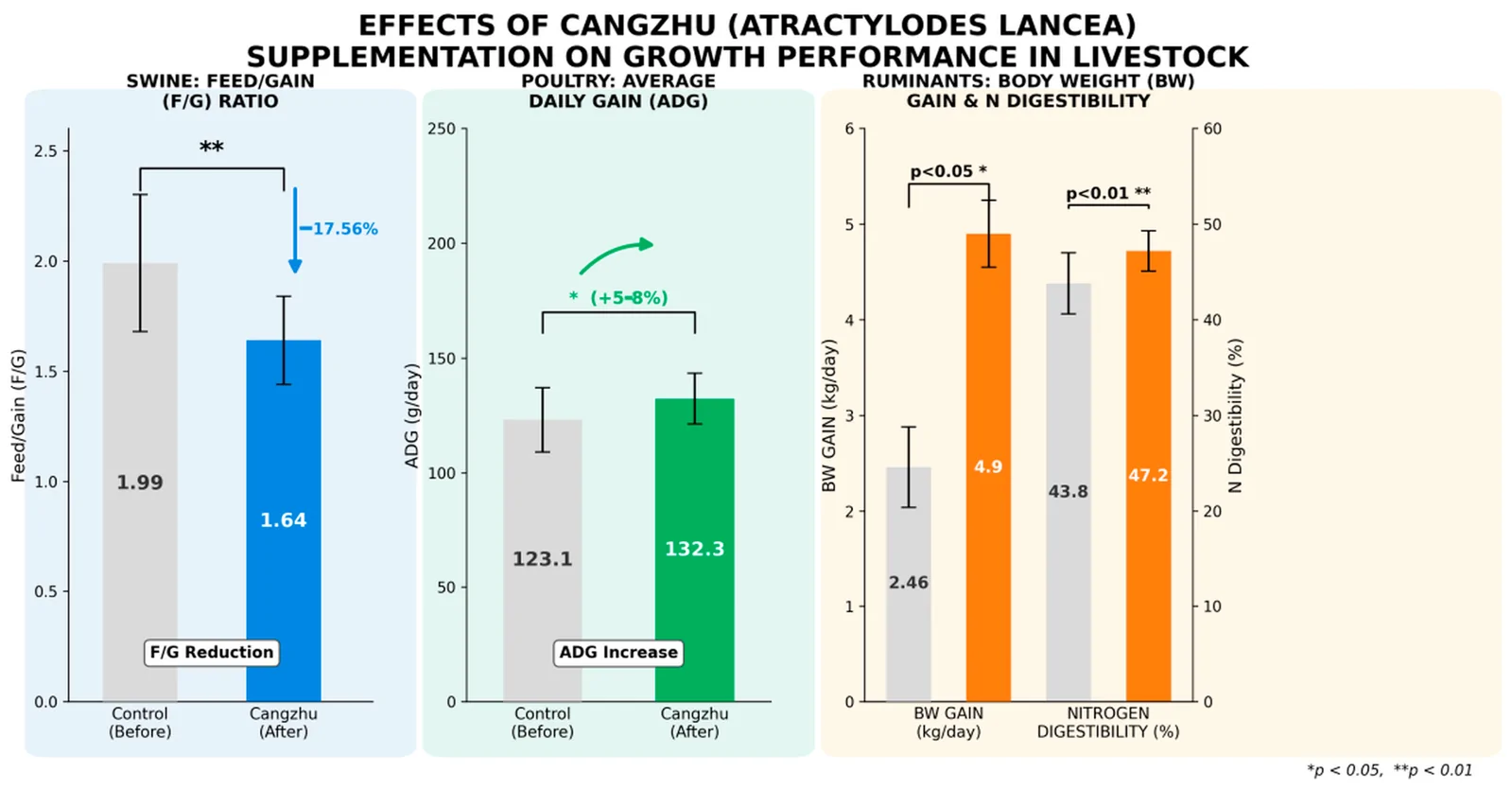
Comparative growth performance improvements from Atractylodes-based supplementation in three major livestock species.

**Figure 7:**
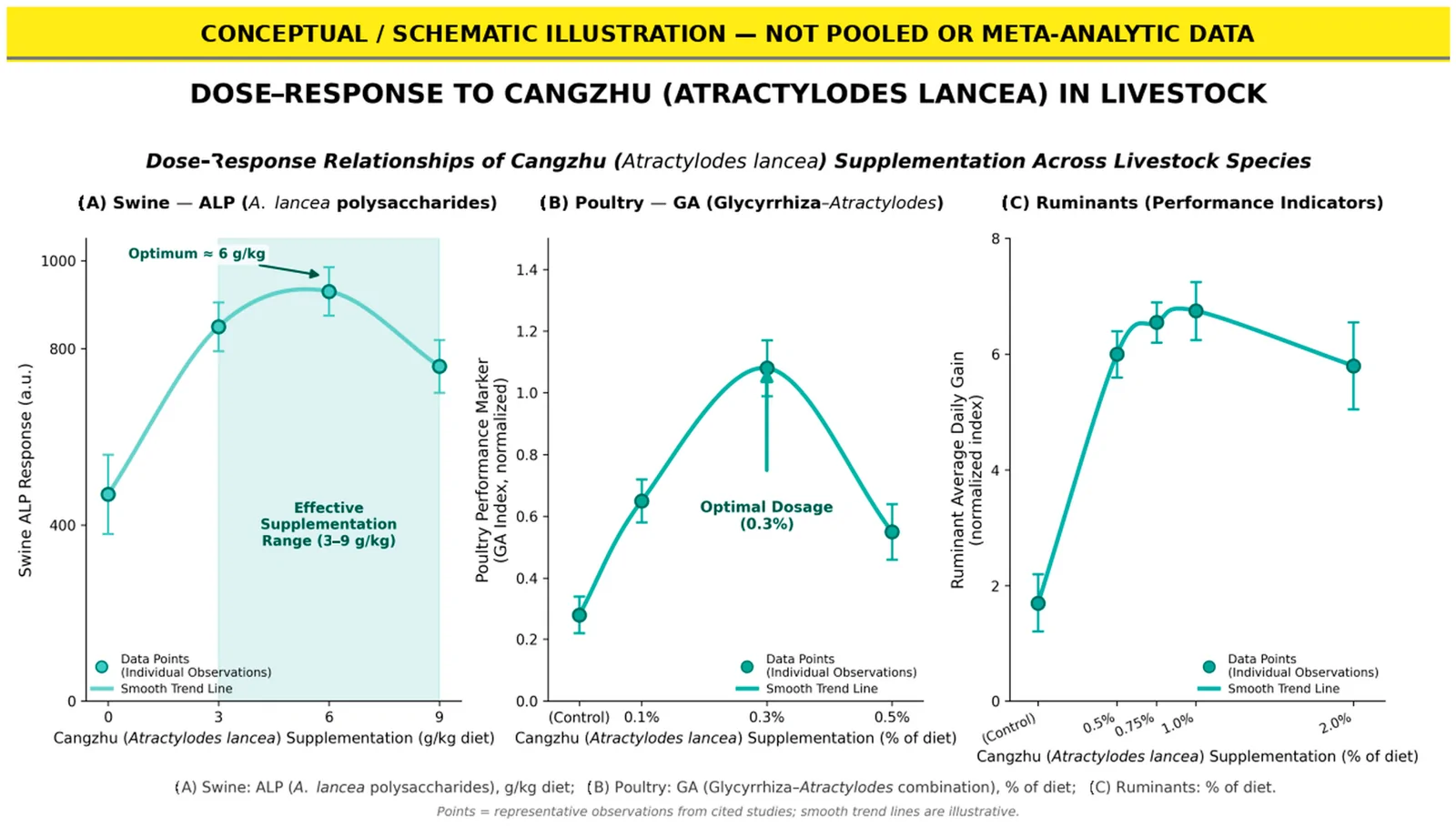
Conceptual, schematic illustration of species-specific dose–response patterns for Atractylodes supplementation.

The quantifiable performance gains shown in significant livestock species are the best way to understand Cangzhu’s translational value as a phytobiotic. Figure 6 offers a comparative summary of the growth performance benefits reported in the individual cited studies, while Figure 7 provides a conceptual illustration of the reported effective inclusion ranges. Both figures are schematic syntheses of single studies; no pooling or meta-analysis was performed (Section 2), and the Atractylodes species/formulation behind each data point is specified in Table 2, Table 3 and Table 4.

Swine: F/G reduction of 17.56% reported by Lin et al. [35] with an Atractylodes-containing herbal formula; Poultry: ADG increase of 5–8% and F/G reduction with GA combination at 0.3% [19]; ruminants: body weight gain increase (*p* = 0.03) and nitrogen digestibility improvement with TCM mixture at 2% [36,37]. The authors’ synthesis was based on Gao et al. [13], Lin et al. [35], Tian et al. [19], and Liang et al. [36,37]. This panel is a schematic summary of the cited single studies (which used different Atractylodes species/formulations; see Table 2, Table 3 and Table 4); error bars and positions are illustrative, and no between-study pooling was performed.

This figure is illustrative only and serves as a conceptual synthesis across heterogeneous studies; it is not a data-derived dose–response analysis, and no pooling or meta-analysis was performed. The inclusion levels plotted are the experimental study ranges reported in the original investigations and must NOT be interpreted as standardized nutritional or therapeutic dose recommendations. Symbols denote representative observations from the cited studies, positioned within their reported inclusion ranges, and the smooth trend lines serve solely to convey the reported nonlinear, dose-dependent character of the responses; both axes are schematic (arbitrary units). Ruminants: *A. lancea* and Atractylodes-containing TCM formulations at 0.5–2.0% inclusion, supported by in vitro rumen fermentation [31] and in vivo formulation trials [13,36,37]; the effective range for pure *A. lancea* still requires in vivo dose–response confirmation in ruminants. Swine: *A. lancea* polysaccharides (ALP) at 3, 6, and 9 g/kg feed showed the highest range of efficacy for minimizing diarrhea and encouraging growth [13]. Poultry: the Glycyrrhiza uralensis-*Atractylodes macrocephala* (GA) combination was tested at 0.1%, 0.3% (optimal), and 0.5%, with the best performance at 0.3% [19]. The necessity of species-specific optimization is highlighted by the nonlinear and dose-dependent nature of these effects; given the heterogeneous formulations and study designs underlying each range, the curves must not be read as quantitative dose–response functions or as recommended inclusion levels. The authors’ synthesis was based on Gao et al. [13], Tian et al. [19], Xu et al. [31], and Liang et al. [36,37]; the schematic was conceived and drawn by the authors for this review, and no elements were reproduced directly from copyrighted material.

### 5.1. Swine Production

In intensive animal agriculture, swine production during the post-weaning transition (21–28 days) is one of the most physiologically demanding stages. Following the removal of AGP, post-weaning stress syndrome, which is marked by villous atrophy, crypt hyperplasia, reduced barrier function, dysbiosis, and a high frequency of diarrhea, has become increasingly prevalent [32]. Evidence summarized in Section 4.5 largely from the related species *A. macrocephala* and Atractylodes-containing formulations [13,33,34] indicates benefits on growth, immunoglobulins, and intestinal morphology in early-weaned pigs, while dedicated in vivo trials with pure *A. lancea* remain a priority gap. In growing-finishing pigs over a 120-day period, a Chinese herbal formulation including Atractylodes decreased F/G by 17.56% relative to negative controls after 120 days [35]. In cyclophosphamide-immunosuppressed mice, Wang et al. [14] showed that FALP at 1.2 g/kg/day enhanced immunological indices and intestinal barrier indicators (preclinical rodent model; Table 2).

**Table 2:** Summary of studies evaluating Atractylodes-based interventions in pigs and preclinical pig models, with explicit identification of the Atractylodes species/formulation and evidence tier.

Study	Atractylodes Species/Formulation	Animal Model (Evidence Tier)	Treatment & Dose	Key Outcomes (Statistics Where Reported)	Mechanistic Highlights
Li et al. [33]	*A. macrocephala* polysaccharides (extrapolated evidence)	Early-weaned piglets (livestock, in vivo)	*A. macrocephala* polysaccharides	↑ ADG; ↑ antibodies; ↓ diarrhea (directional effects; statistics NR)	Lymphocyte proliferation; antioxidant
Wang et al. [34]	Atractylodes-containing fermented formula (species not specified)	Early-weaned piglets (livestock, in vivo)	0.1–0.3%	Dose-dependent ↑ IgA, IgG, IgM (directional; statistics NR)	Hepatic antioxidant/anti-inflammatory status
Lin et al. [35]	Atractylodes-containing herbal formula (species not specified)	Grow-finish pigs, 120 d (livestock, in vivo)	Chinese herbal formula	↓ F/G by 17.56% vs. negative control	↑ VH/CD; ↑ nutrient digestibility
Wang et al. [14]	*A. lancea* polysaccharides (FALP/SALP)	Cyclophosphamide-immunosuppressed mice (preclinical rodent model)	SALP & FALP, 1.2 g/kg/day	FALP superior: ↑ WBC, sIgA (directional; statistics NR)	TLR4; ↑ GPR43; ↑ *Lactobacillus*

VH = villus height; CD = crypt depth; FALP = bran-fried *A. lancea* polysaccharides; SALP = raw ALP; NR = inferential statistics not reported (or not retrievable) in the source. Only a subset of the summarized studies used pure *A. lancea*; rows derived from *A. macrocephala* or multi-herb formulas are flagged in the species column and should be interpreted as extrapolated evidence. ↑ = increase and ↓ = decrease relative to the corresponding control group, as reported in the source studies; NR = not reported.

Critical synthesis (swine): For swine, the evidence base is strongest for the related species *A. macrocephala* and for multi-herb or fermented Atractylodes formulations, which consistently report favorable directional effects on growth, antibody titres, and intestinal morphology in early-weaned and growing-finishing pigs [33,34,35]. This evidence is nonetheless limited: most reported outcomes are directional with inferential statistics not reported, the active Atractylodes species is frequently unspecified, and two of the four studies (Li et al. [33] and Wang et al. [34]) do not use *A. lancea* at all. Discrepancies are therefore attributable primarily to differences in formulation (single herb vs. multi-herb), dose (0.1–0.3% vs. an undefined herbal-formula concentration), and pig category (early-weaned piglets vs. growing-finishing pigs) rather than to any biological inconsistency intrinsic to *A. lancea*. Direct evidence from pure *A. lancea* in swine in vivo is currently confined to a single preclinical rodent model (Wang et al. [14]); consequently, *A. lancea*-specific performance claims in pigs remain extrapolated and require dedicated, dose-capture trials in the target species.

### 5.2. Poultry Production

Modern broiler genotypes are more vulnerable to *Eimeria* spp. (coccidiosis) and *Clostridium perfringens* (necrotic enteritis) due to their high metabolic rates and increased oxidative stress [27]. Direct poultry trials with *A. lancea* remain scarce; most polysaccharide evidence in poultry currently derives from the related *A. macrocephala* (Baizhu) [13], and interspecific extrapolation should therefore be made cautiously. The glycyrrhiza uralensis–*Atractylodes macrocephala* combination (GA) at 0.3% was shown by Tian et al. [19] to significantly increase ADG from days 29–84 and decrease F/G, elevate thymus and bursa indices, increase IgA, IgM, and IL-10 while decreasing IL-2 and TNF-α, and upregulate ZO-1 and CLDN1 mRNA in the jejunum. Metabolomic profiling revealed enhanced butyric acid levels in the cecal microbiota, which also exhibited decreased abundance of Desulfovibrio and Campylobacter and increased abundance of Butyricicoccus and Muribaculaceae. Liu et al. [38] found that a TCM additive containing Atractylodes at 400 mg/kg increased egg production, improved GSH-Px, T-AOC, and SOD, and elevated IL-2, IFN-γ, IgA, IgM, and IgG in laying hens. Sun et al. [39] found that a designed herbal formula containing *A. macrocephala* at 1.0% increased body weight and decreased FCR, with increased beneficial Bacteroides and Faecalibacterium (Table 3; evidence tier and species identity are specified for each study).

**Table 3:** Summary of Atractylodes-based interventions in poultry, with explicit identification of the Atractylodes species/formulation and evidence tier.

Study	Atractylodes Species/Formulation	Animal Model (*n*)	Treatment & Dose	Key Outcomes (Statistics Where Reported)	Mechanistic Highlights
Tian et al. [19]	*A. macrocephala* in GA combination (extrapolated evidence)	Broiler chickens (*n* = 360, 4 groups)	GA 0.1%, 0.3%, 0.5%	↑ ADG 5–8% (d 29–84); ↓ F/G; ↑ IgA, IgM, IL-10; ↓ IL-2, TNF-α	↑ ZO-1, CLDN1; ↑ cecal butyrate
Sun et al. [39]	*A. macrocephala*-containing herbal formula (extrapolated evidence)	Broiler chickens (*n* = 240, 3 groups)	Herbal formula 1.0%	↑ BW; ↓ FCR (directional; statistics NR)	↑ Bacteroides, Faecalibacterium
Liu et al. [38]	TCM additive containing Atractylodes (species not specified)	Laying hens (*n* = 180, 3 groups)	TCM additive, 400 mg/kg	↑ egg production/quality; ↑ GSH-Px, T-AOC, SOD; ↑ IL-2, IFN-γ, IgA, IgM, IgG (directional; statistics NR)	Antioxidant and immune enhancement
Hu et al. [40]	*A. macrocephala* polysaccharides (extrapolated evidence; in vitro)	In vitro (poultry cells)	*A. macrocephala* polysaccharides	↑ immunomodulation (in vitro only; statistics NR)	In vitro study
Wang et al. [34]	*A. lancea* extract	Broilers (*n* = 300, 5 groups)	*A. lancea* extract 0.5–2.0%	↑ growth; ↓ diarrhea (directional; statistics NR)	Anti-inflammatory

↑ = increase and ↓ = decrease relative to the corresponding control group, as reported in the source studies. GA = G. uralensis–*A. macrocephala* combination; NR = inferential statistics not reported (or not retrievable) in the source. Direct trials with pure *A. lancea* in poultry remain scarce; rows flagged as extrapolated derive from *A. macrocephala* or mixed herbal formulas.

Critical synthesis (poultry): For poultry, the strongest and most internally consistent evidence derives from the *A. macrocephala*-containing GA combination (Tian et al. [19]), which reported significant, dose-modulated improvements in growth, immune indices, and gut-barrier markers at 0.3%. This finding is qualitatively supported by the *A. macrocephala*-containing formula of Sun et al. [39] and by the Atractylodes-containing TCM additive of Liu et al. [38] in laying hens, although the latter two report only directional effects. Agreement is therefore strongest for anti-inflammatory, immune-enhancing, and barrier-supporting effects of Atractylodes-containing formulations. Discrepancies arise mainly from differences in formulation (*A. macrocephala* vs. unspecified Atractylodes species), dose (0.1–0.5% in broilers vs. 400 mg/kg in layers), and production objective (broiler growth vs. egg production), rather than from conflicting biological findings. The only study reporting pure *A. lancea* in poultry (Wang et al. [34]) is directionally supportive but lacks reported statistics and dose resolution, so direct *A. lancea* evidence in poultry remains limited and should be interpreted cautiously pending confirmatory trials.

### 5.3. Ruminant Production

The rumen ecosystem supplies 70–80% of the host energy and 60–80% of the amino acid requirements via VFAs and microbial protein [41]. Gao et al. [13] note that 0.75% *A. lancea* enhances rumen fermentation, increases microbial protein and cellulose degradation, and promotes bacterial protein synthesis. Liang et al. [36] demonstrated that a TCM combination (Astragalus root, Angelica root, and Atractylodes rhizome) at 2% significantly increased sheep body weight gain (*p* = 0.03), total VFA concentration, and acetate generation (*p* = 0.04). Significantly higher N intake (*p* < 0.01), better N digestibility (*p* = 0.02), lower fecal N excretion (*p* = 0.02), and higher microbial N supply (*p* < 0.01) were also supported by a follow-up investigation by Liang et al. [37] (Table 4). As noted in Section 4.4, the ruminant evidence base is anchored by in vitro batch-culture data (Xu et al. [31]) and in vivo trials of multi-herb TCM mixtures containing Atractylodes rather than controlled dose–response trials with pure *A. lancea*; *A. lancea*-specific dosing in ruminants should therefore be extrapolated cautiously (Table 4, evidence-tier column).

**Table 4:** Summary of ruminant studies incorporating Atractylodes, with explicit identification of the material, model, and evidence tier.

Study	Atractylodes Material	Model & Design (Evidence Tier)	Treatment	Key Outcomes (Statistics Where Reported)	Metabolic/Rumen Effects
Xu et al. [31]	Rhizoma Atractylodis essential oil	In vitro rumen batch culture, Jinjiang yellow cattle (in vitro only)	Rhizoma Atractylodis essential oil	↑ fermentation; ↑ nutrient degradability (directional; statistics NR)	↑ microbial protein; ↑ cellulose degradation
Liang et al. [36]	Atractylodes rhizome in TCM mixture (with Astragalus, Angelica)	Sheep, 35 d crossover (livestock, in vivo)	TCM mixture 2%	↑ BW gain (*p* = 0.03)	↓ rumen pH; ↑ total VFA; ↑ acetate (*p* = 0.04)
Liang et al. [37]	Atractylodes rhizome in TCM mixture (with Astragalus, Angelica)	Sheep, 21 d crossover (livestock, in vivo)	TCM mixture 2%	↑ N intake (*p* < 0.01); ↑ N digestibility (*p* = 0.02); ↓ fecal N (*p* = 0.02)	↑ purine derivatives; ↑ microbial N supply (*p* < 0.01)

↑ = increase and ↓ = decrease relative to the corresponding control group, as reported in the source studies. NR = inferential statistics not reported (or not retrievable) in the source; the row from Xu et al. [31] is in vitro evidence only. TCM combines Atractylodes rhizome, Astragalus root, and Angelica root.

Critical synthesis (ruminants): The ruminant evidence is the thinnest of the three livestock groups and is almost entirely derived from either in vitro batch-culture systems (Xu et al. [31]) or multi-herb TCM mixtures in which Atractylodes is only one of several active components (Liang et al. [36,37]). Where strong, methodologically sound evidence exists (the two crossover trials of Liang et al.), the reported protein-energy and nitrogen-economy benefits are internally consistent and statistically supported, at a fixed 2% inclusion of the mixture. Agreement across studies is therefore limited to the general direction of rumen-fermentation and N-utilization improvement; direct comparisons are complicated by marked differences in model system (in vitro vs. sheep), formulation (essential oil vs. TCM mixture), and the absence of any pure *A. lancea* dose–response trial in ruminants. Consequently, species-specific effects cannot yet be attributed specifically to *A. lancea*, and the single *A. lancea* observation (0.75% reported by Gao et al. [13]) must be regarded as extrapolated pending in vivo confirmation.

## 6. Sustainability, One Health, and Broader Implications

### 6.1. AMR Mitigation

Cangzhu provides a mechanism-based alternative approach for the control of pathogens. Atractylodin had significantly higher potency (MIC 0.02–0.1 mg/mL against *E. coli* and S. aureus), while the MIC values of *A. macrocephala* essential oil against major livestock pathogens ranged from 0.5 to 2.0 mg/mL [21]. The antibacterial mechanism involves the disruption of the protein structure of the bacterial cell membrane, a physical mechanism that has been proposed to reduce selective pressure for resistance compared with a single-target antibiotic; this proposition is mechanistically reasonable but has not been experimentally demonstrated for *A. lancea* and therefore constitutes a hypothesis to be tested rather than an established property. Novel sesquiterpenoids and polyacetylenes from *A. lancea* have also been reported to show potent anti-MRSA activity (MIC 6.25–20.00 μg/mL) [13]. We emphasize, however, that these observations constitute direct antimicrobial activity demonstrated in vitro; experimentally demonstrated AMR mitigation (e.g., reduced resistance-gene transfer or reduced pathogen loads under field conditions) has not yet been shown for *A. lancea* and remains a research priority. In summary, the currently available data establish antimicrobial activity of *A. lancea* constituents that warrants investigation for a potential role in reducing pathogen pressure and, consequently, antibiotic use, rather than a proven reduction in resistance risk. Furthermore, *A. lancea* material has been reported to directly adsorb aflatoxins, which has been suggested to provide simultaneous protection against mycotoxicosis [13]; however, the underlying mechanism, binding efficacy, and performance relative to established mycotoxin binders (e.g., hydrated sodium calcium aluminosilicates) have not been characterized, so this putative benefit should be considered preliminary pending dedicated mycotoxin-binding assays.

### 6.2. Environmental Sustainability

Life cycle assessment studies have demonstrated that interventions that improve feed conversion efficiency through enhanced gut health reduce the carbon footprint of animal products by 8–15%, with each 0.1-unit FCR improvement associated with 3–5% reductions in GHG emissions [42]. These figures derive from general livestock LCA relationships [42] rather than from Cangzhu-specific life-cycle assessments; extrapolating them to Cangzhu is an inference and should be read as a potential, order-of-magnitude estimate rather than a measured outcome. Given the reported FCR improvements with Cangzhu or Atractylodes-based supplementation (F/G reductions of 5–18% in swine, [35]; 3–8% in poultry, [19,39]), the environmental benefits of its widespread adoption are plausible and potentially substantial. In ruminants, enhanced N utilization translates to reduced urinary and fecal N excretion [36,37].

### 6.3. One Health Integration

**Figure 8:**
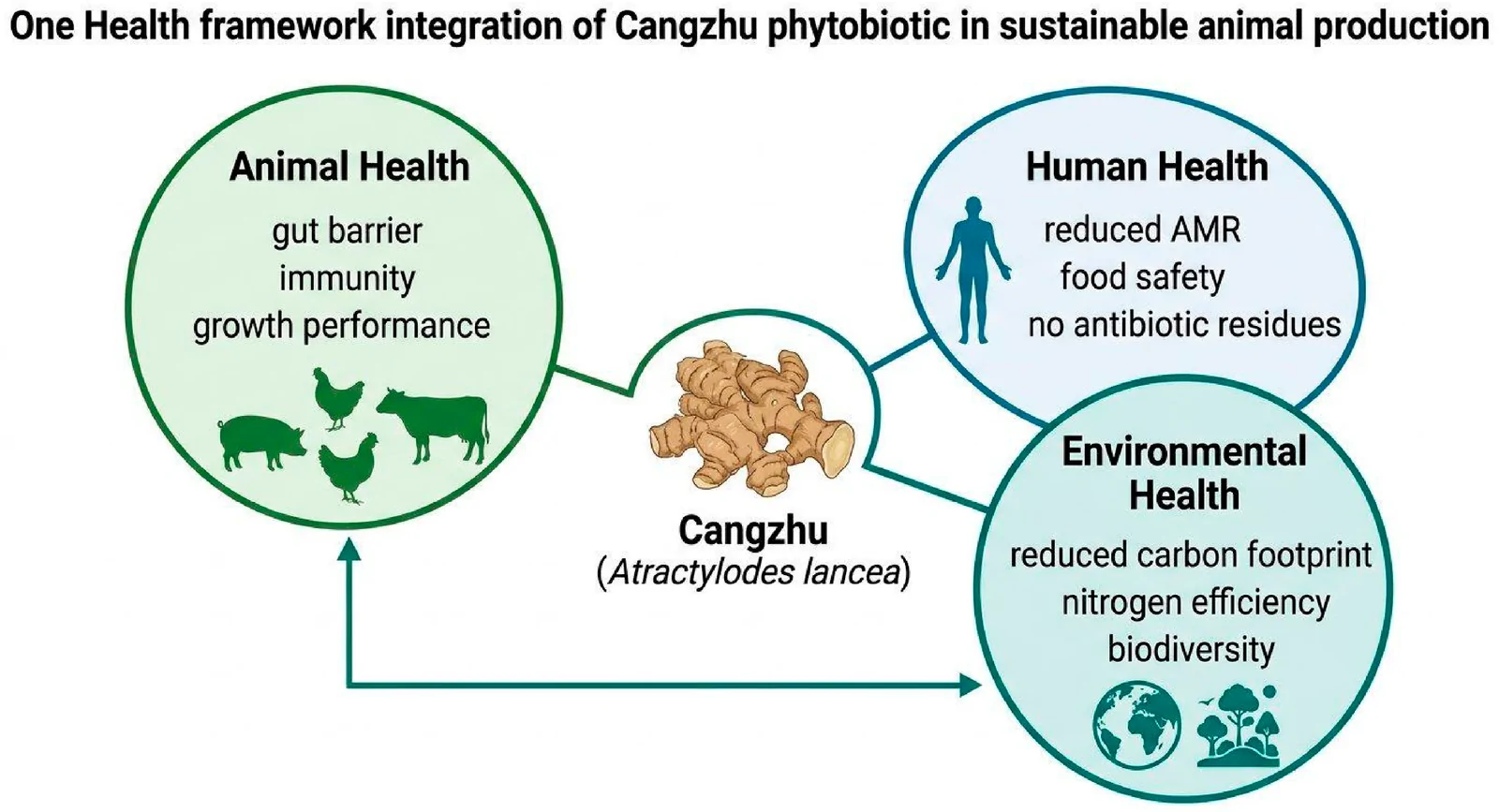
How Cangzhu (*Atractylodes lancea*) is included into the One Health framework for precision antibiotic-free animal production.

The connection of environmental, animal, and human health is acknowledged by the One Health concept (Figure 8). Within this framework it is important to distinguish between animal gut-health outcomes that have been empirically demonstrated (improved growth performance, immune competence, and gut-barrier function) and the human-health and environmental benefits that follow from them, which are currently potential and inferred rather than directly measured (Figure 8). According to Gao et al. [13] and Gerber et al. [42], Cangzhu’s mechanisms support the following three pillars: (i) Animal Health: improved growth performance, immune competence, and gut barrier function; (ii) Human Health: reduced antibiotic use has the potential to lower AMR selection pressure and antibiotic residues in animal products, although this downstream benefit has not yet been measured in Cangzhu-supplemented systems; and (iii) Environmental Health: improved feed efficiency and N economy are expected to reduce environmental impact while *A. lancea* cultivation may promote agricultural biodiversity. These environmental benefits are inferential extrapolations from general livestock relationships rather than Cangzhu-specific measurements.

Cangzhu phytobiotic supplementation connects three interrelated domains: Animal Health (gut barrier integrity, immune competence, and growth performance), Human Health (AMR mitigation, food safety, and reduced antibiotic residues), and Environmental Health (reduced GHG emissions, improved nitrogen efficiency, and agricultural biodiversity). The animal-health outcomes shown in the central domain are the most directly supported by the current evidence; the human-health and environmental-health benefits are reasonable, mechanistically supported inferences that follow from these animal outcomes and are expected rather than already demonstrated for Cangzhu. The authors’ synthesis was based on Ogbuewu et al. [9], Gerber et al. [42], and Gao et al. [13]. The figure was designed and drawn specifically for this review from the authors’ synthesis of these sources; no elements were reproduced directly from copyrighted material.

## 7. Challenges, Knowledge Gaps, and Future Research Directions

### 7.1. Standardization and Quality Control

Strict quality criteria are needed to convert research-grade phytobiotics into commercial feed additives. Atractylodin is the only pharmacopoeial index; however, network pharmacology proposes atractylon, hinesol, and β-eudesmol as more functionally relevant Q-markers [38]. Additional barriers to standardization include batch-to-batch variability caused by genetic, geographic, seasonal, and processing factors [18], the lack of stability data under typical feed processing and storage conditions, as well as the absence of proven high-throughput analytical methods suitable for feed mill settings.

### 7.2. Dose–Response Optimization

The effects of Cangzhu are dose-dependent and frequently nonlinear (Figure 7), with different species having different effective inclusion rates reported in the cited literature: 0.5–2% in ruminants (in vitro and TCM-formulation trials) [13,36], 3–9 g/kg ALP in swine [13], and roughly 0.3% GA in poultry [19]. Because these ranges derive from heterogeneous formulations, species, and study designs (Table 2, Table 3 and Table 4), they should be viewed as starting points for systematic experimentation rather than validated recommendations. It must be emphasized that these inclusion levels are experimental study ranges reported in the primary investigations and are not standardized nutritional or therapeutic dose recommendations for any livestock species. They cannot be applied directly to commercial diets; dose selection for any production setting requires species- and stage-specific, dose–response validation carried out under controlled conditions regardless of the values plotted in Figure 7. In addition to assessing synergistic interactions with other phytobiotics, organic acids, probiotics, or enzymes, systematic dose–response experiments across production stages, genotypes, and management methods are required.

### 7.3. Gut Microbiota–Metabolite–Host Axes

Although compositional changes in gut microbiota after Cangzhu supplementation have been reported by 16S rRNA studies [15,19], the functional implications and causative roles of particular taxa are yet unknown. To identify modulated microbial metabolic pathways, characterize altered metabolites (SCFAs, bile acids, and tryptophan metabolites), establish causality through fecal microbiota transplantation in gnotobiotic models, and clarify the pharmacokinetics and microbial biotransformation of Cangzhu constituents, multi-omics integration (metagenomics, metatranscriptomics, and metabolomics) is necessary [30].

### 7.4. Large-Scale Field Validation and Economic Analysis

Most studies on the efficacy of Cangzhu have been conducted under controlled research conditions. Translation to commercial practice requires large-scale, multi-site field trials with rigorous economic analyses accounting for raw material costs, processing, feed incorporation, improved performance value, reduced therapeutic antibiotic expenditure, and potential market premiums for antibiotic-free products [8,11].

### 7.5. Novel Delivery Systems

The volatility and oxidation susceptibility of Cangzhu sesquiterpenoids pose practical challenges. Advanced technologies such as microencapsulation, nanoencapsulation, solid lipid nanoparticles, and enteric coating offer solutions by protecting bioactives during processing, masking unpalatable flavors, and enabling targeted gastrointestinal release [8,9]. Fermented Cangzhu products, which demonstrate enhanced bioactivity through microbial pre-digestion, simultaneously address challenges of stability, palatability, and bioavailability [13].

## 8. Conclusions

Cangzhu (*Atractylodes lancea*) constitutes a promising class of phytobiotics with genuine potential to reduce reliance on antibiotics in livestock production as part of integrated, gut-health-centered feeding strategies. The evidence that Cangzhu and its bioactive components provide gut-protective benefits through a coordinated, multi-target mechanism is summarized in this review. Tight junction protein upregulation, concurrent suppression of the NF-κB and MAPK inflammatory pathways, activation of the Nrf2-Keap1 antioxidant pathway, partial normalization of the gut microbial community, stimulation of mucosal immunity by TLR4-dependent and SCFA-GPR43 signaling, and direct antimicrobial activity that warrants investigation for a potential role in reducing pathogen pressure and antibiotic use are some of the mechanisms that improve the intestinal epithelial barrier. As summarized in Figure 5, Figure 6 and Figure 7 and Table 2, Table 3 and Table 4, these pathways frequently correlate with gains in livestock productivity, including increased growth rates, higher feed efficiency, decreased incidence of diarrhea, and strengthened immune competence across swine, poultry, and ruminant systems. Nevertheless, a substantial portion of the mechanistic evidence derives from rodent and in vitro models and from the related *A. macrocephala* or multi-herb formulations, and direct, species-specific livestock trials with pure *A. lancea* remain limited for several endpoints; this distinction is now made explicit throughout the review. International harmonization of quality standards, systematic dose–response trials, thorough multi-omics elucidation of microbiota-host interactions, large-scale field trials with economic analyses, and sophisticated formulation technology are all necessary for the shift from promising research to commercial adoption. When incorporated into precision animal nutrition programs under the guidance of species-specific optimal dosing, synergistic formulation, and ongoing quality monitoring and subjected to standardized, commercial-scale, species-specific validation Cangzhu holds substantial potential to contribute to sustainable, antibiotic-reduced animal agriculture. This transition from traditional knowledge to cutting-edge feed technology is a prime example of the fruitful dialog between ethnopharmacology and current animal science that will influence livestock production in the future.

## Figures and Tables

**Table 1:** *Atractylodes lancea* rhizomes’ principal bioactive components and the basis on which their content is reported, with explicit source-species and evidence attribution.

Compound Class	Representative Compounds	Relative Content	Reported Basis	Key Biological Activities	Analytical Method	Source Species/Evidence Attribution
Sesquiterpenoids	Atractylone	1.32–39.22%	Relative % of the essential-oil fraction (peak-area normalization)	Anti-inflammatory, antioxidant	GC-MS, GC-FID	[Direct: *A. lancea*]
Sesquiterpenoids	β-Eudesmol	0.81–27.70%	Relative % of the essential-oil fraction (peak-area normalization)	Anti-angiogenic, antimicrobial	GC-MS	[Direct: *A. lancea*]
Sesquiterpenoids	Hinesol	3.52–5.50%	Relative % of the essential-oil fraction (peak-area normalization)	Antibacterial, anti-inflammatory	GC-MS	[Direct: *A. lancea*]
Sesquiterpenoids	Atractylenolide I	~0.45%	Relative % of the assayed extract/fraction, as reported	NF-κB inhibition, TNF-α/NO suppression	UPLC-MS/MS	[Direct: *A. lancea*; in vitro]
Sesquiterpenoids	Atractylenolide II	~5.07%	Relative % of the assayed extract/fraction, as reported	MAPK pathway modulation	UPLC-MS/MS	[Direct: *A. lancea*; in vitro]
Sesquiterpenoids	Atractylenolide III	Trace–variable	Relative % of the assayed extract/fraction, as reported	Nrf2 activation, immunomodulation	LC-Triple TOF-MS/MS	[Direct: *A. lancea*; rodent model]
Polyacetylenes	Atractylodin, acetylatractylodinol	0.30–6.22%	Relative % of the essential-oil/extract fraction, as reported	Broad-spectrum antibacterial (MIC 0.02–0.1 mg/mL); anti-inflammatory; gut motility	GC-MS, LC-MS/MS	[Direct: *A. lancea*; *A. macrocephala* (Wu et al. [21])—extrapolated]
Polysaccharides	*A. lancea* polysaccharides (ALP)	Variable	Extraction yield (% of dry rhizome weight); highly method-dependent	Immunomodulation, gut barrier repair, prebiotic	Phenol-sulfuric acid	[Direct: *A. lancea*]
Flavonoids & phenolics	Ferulic acid, 4-methylumbelliferone	Trace	Relative % of the assayed extract, as reported	PI3K-AKT inhibition, antioxidant	UPLC-MS/MS	[Direct: *A. lancea*; in vitro]

Gao et al. [13], Wu et al. [21], Guo et al. [20], and Zhang et al. [18] provided the data. Note: relative-content values are indicative ranges compiled from the cited sources and are expressed on different bases (essential-oil fraction, extract/fraction, or dry-rhizome extraction yield); they are therefore not directly comparable across rows. The basis of each value is specified in the “Reported basis” column, and readers should consult the original publications for exact quantification conditions. Atractylodin is a polyacetylenic compound and is therefore listed under polyacetylenes; the sesquiterpenoid rows comprise the non-polyacetylene constituents of the essential-oil fraction. Composition data refer to *A. lancea* unless indicated otherwise; rows for which activity data also derive from *A. macrocephala* essential oil [21] are flagged in the species/source column.

## Data Availability

No new data were created or analyzed in this study. Data sharing is not applicable to this article.

## Appendix A

A thorough overview of the molecular pathways, active ingredients, and physiological effects of Cangzhu-mediated gut protection in the species studied to date is given in Table A1. Evidence for the pathways below derives variously from rodent, in vitro, and livestock studies; study-level evidence tiers are specified in Table 2, Table 3 and Table 4.

**Table A1:** Comprehensive summary of Cangzhu-mediated gut-protective mechanisms across experimental models. ALP, *A. lancea* polysaccharides; EO, essential oil.

Pathway/Target	Active Constituent(s)	Experimental Model/Species	Effect	Molecular Consequence	Physiological Outcome
NF-κB	Atractylenolide I, III	In vitro macrophages; rodent models	↓ Inhibition	↓ p65 translocation; ↓ IκBα-P; ↓ TNF-α, IL-1β, IL-6	Reduced intestinal inflammation
MAPK (p38, ERK, JNK)	Atractylenolide II	In vitro cell models; rodent models	↓ Phosphorylation	↓ AP-1; ↓ COX-2, iNOS	Reduced inflammatory mediators
Nrf2-Keap1	ALP, triterpenoids	Rodent models (e.g., LPS-exposed mice)	↑ Activation	↑ Nrf2 nuclear; ↑ HO-1, NQO1, GSTA1, GSTM1	Enhanced antioxidant defense
Tight junctions	ALP	Rodent/in vitro; broiler jejunum via *A. macrocephala* formula	↑ Expression	↑ ZO-1, Occludin, Claudin-1	Reinforced intestinal barrier
TLR4	ALP	Cyclophosphamide-immunosuppressed mice	↑ Activation	↑ Macrophage activity; ↑ GPR43	↑ Mucosal immunity; ↑ sIgA
Gut microbiota	ALP, essential oils	Antibiotic-induced dysbiosis mouse model; broiler cecum	Modulation	↑ *Lactobacillus*, Faecalibaculum; ↓ *E. coli*	Partially normalized microbial composition; ↑ SCFA
Bacterial membrane	Atractylodin, EO	In vitro antimicrobial assays	Disruption	Protein disruption; cell leakage	Bactericidal (MIC 0.02–2.0 mg/mL)
Mucin secretion	*A. lancea* extract	Rodent/in vitro models	↑ Stimulation	↑ Goblet cells; ↑ MUC2	Thickened mucus layer
Rumen fermentation	*A. lancea* rhizome	In vitro rumen batch culture (Jinjiang yellow cattle)	↑ Enhancement	↑ VFA; ↑ acetate; ↑ microbial protein	↑ N utilization

Note: ↑, increase/upregulation/activation; ↓, decrease/downregulation/inhibition.
